# Cellular Uptake of Hybrid PLGA-Lipid Gadolinium Nanoparticles Functionalized for Magnetic Resonance Imaging of Pancreatic Adenocarcinoma Cells

**DOI:** 10.1021/acsnanoscienceau.5c00010

**Published:** 2025-04-24

**Authors:** Alessandro Amaolo, Hanieh Sadeghi, Carla Carrera, Sergio Padovan, Fabio Carniato, Enza Di Gregorio, Giuseppe Ferrauto

**Affiliations:** † Molecular Imaging Center, Department of Molecular Biotechnologies and Health Sciences, University of Torino, Via Nizza 52, Torino 10126, Italy; ‡ Dipartimento di Scienze e Innovazione Tecnologica, Università degli Studi del Piemonte Orientale, Via Teresa Michel 11, Alessandria 15121, Italy

**Keywords:** magnetic resonance imaging, molecular imaging, nanoparticles, pancreatic cancer, PLGA, theranostic

## Abstract

Pancreatic adenocarcinoma (PDAC) presents significant diagnostic challenges, necessitating improved imaging techniques. Here, we develop hybrid poly­(lactic-*co*-glycolic acid) (PLGA)-phospholipid nanoparticles (NPs) loaded with gadolinium (Gd) chelates and functionalized with albumin, adenosine, or glutamine to boost their internalization in PDAC cells and increase the detectability by magnetic resonance imaging (MRI). Gd-PLGA NPs were synthesized using an *oil-in-water* emulsion solvent extraction method and incorporating DSPE-PEG(2000)­methoxy and DPPE-PEG(2000) *N*-Hydroxysuccinimide (NHS) for surface functionalization with albumin, adenosine, or glutamine. NPs were characterized by dynamic light scattering for particle size and ζ potential measurements, in addition to ^1^H NMR and proton nuclear magnetic relaxation dispersion to assess relaxivity and contrastographic properties, and stability studies were conducted in both HEPES-buffered saline and human serum. Reported studies demonstrated that all the preparations display a good stability, a hydrodynamic diameter lower than 200 nm, and a slight negative surface charge, with good potential for applications in cells and in vivo. In vitro studies on MiaPaca2 and Panc1 cell lines confirmed that functionalized NPs display higher cellular uptake and stronger MRI signal enhancement than unconjugated controls, with albumin-PLGA-lipid NPs leading to the greatest uptake. Our findings highlight a promising route toward a more sensitive, targeted MRI of PDAC, calling for in vivo studies to assess diagnostic potential and therapeutic applications.

## Graphical abstract

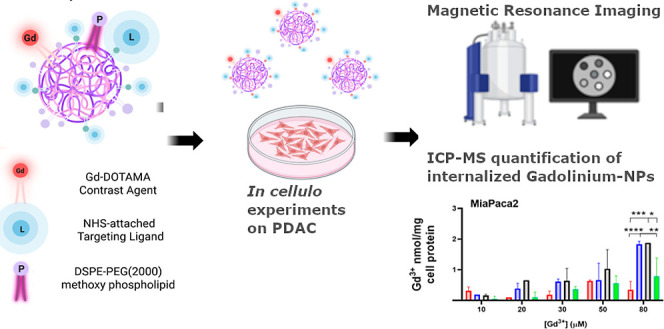

## 1 Introduction

The major challenges in pancreatic cancer are early diagnosis and the establishment of standardized international guidelines in assessing suspicious pancreatic masses. Magnetic resonance imaging (MRI) allows for tumor detection in the early stage with equal sensitivity and specificity as the current gold standard imaging techniques used in clinics.^1^ According to the National Comprehensive Cancer Network pancreatic protocol, the first route of diagnosis when there is clinical suspicion of cancer is multidetector computed tomography, which provides a comprehensive examination of the disease.^2,3^ It includes the administration of intravenous high iodine concentrated contrast agents to maximize the X-ray attenuation differences between the hypovascular tumor and the surrounding pancreatic parenchyma.^4,5^ Despite being considered safe overall, contrast CT often leads to dose-dependent adverse effects, such as nephrotoxicity and exposure to radiation. In addition, there is great variability in the concentration and rate of injection of contrast solution and age, weight, and cardiac output of the examined patient.^3,5^ Therefore, there is a need for a standardized protocol to provide a clearer framework for clinicians to follow, minimizing errors and variability. MRI of the pancreas evaluates the local proton density of tissues within the body with a high spatial resolution without being invasive. Like CT, gadolinium (Gd)-based contrast enhancement agents (GBCAs) are administered at high dose, corresponding to 0.1–0.4 mmol/kg body weight for GBCAs.^6–8^ Furthermore, in specific cases, MRI has an advantage over CT in patients with sensitivities in iodinating contrast and renal impairment and in differentiating iso-attenuating pancreatic lesions.^9^ MR imaging agents work by shortening the longitudinal (*T*_1_) and transverse (*T*_2_) relaxation time of water protons in their vicinity, increasing the contrast of tissues in which they distribute.^10^ Improving their biodistribution to targeted sites could counter the limitations of single molecule-based imaging technologies to augment image quality, enhance temporal resolution, and improve probe sensitivity.^11,12^ Delivering GBCAs encapsulated by nanoparticles (NPs) can overcome this challenge. Protection of these agents from undesirable interactions with biological milieu allows for prolonged plasma *half-life*, hence increasing biodistribution within the tumor, which can be followed in real time, after administration.^13,14^ In vivo uptake of NPs can either passively or actively target the pathology of interest to enhance the accumulation of the active agent at the desired site.^15^ The enhanced permeability and retention (EPR) effect is the mode that best describes this accumulation and is firmly dictated by the permeability of the tumor. However, in vivo uptake and retention of NPs are poor due to the tumor-associated stromal barrier that surrounds the pancreas.

Poly­(lactic-*co*-glycolic acid) (PLGA) is a clinically approved US FDA and European Medicine Agency (EMA) biomaterial used for the fabrication of several NPs.^16^ These polymers are commercially available at different molecular weights and compositions.^16^ PLGA can be part of more complex supramolecular copolymers, formed through noncovalent interactions such as hydrogen bonding, π–π stacking, metal coordination, and host–guest interactions. These systems have gained significant attention due to their dynamic and reversible assembly properties, tunable mechanical strength, self-healing capabilities, and responsiveness to external stimuli such as pH, temperature, and enzymatic activity, making them ideal for biomedical applications, including drug delivery and tissue engineering.^17,18^

A well-studied and applied hybrid structure is the combination of PLGA with PEGylated lipids and DPPE-PEG (2000) NHS). Hybrid PLGA-phospholipid NPs offer several advantages over pure PLGA NPs, primarily due to the incorporation of phospholipids, which enhances their physicochemical and biological properties. The phospholipid layer improves the stability of the NPs by reducing aggregation and providing a more uniform particle size distribution.^19^ Additionally, phospholipids enhance biocompatibility and reduce immune system recognition, leading to prolonged circulation time in the bloodstream.^20^ These hybrid NPs also exhibit improved drug loading efficiency and controlled release profiles, allowing for more effective and sustained drug delivery.^21^ Moreover, the phospholipid component facilitates better interaction with biological membranes, improving cellular uptake and bioavailability of encapsulated therapeutics.^22^ Furthermore, incorporating a phospholipid containing an active group, such as maleimide or carboxyl, enables bioconjugation with targeting ligands, antibodies, or peptides, enhancing selective drug delivery to specific cells or tissues.^23^ As a result, hybrid PLGA-phospholipid NPs provide a versatile and superior drug delivery platform compared to pure PLGA NPs, making them highly valuable for biomedical applications. This hybrid approach holds great potential for advancing nanomedicine by optimizing the performance of PLGA NPs for precision drug delivery applications.

Herein, Gd-loaded poly­(lactic-*co*-glycolic acid) (PLGA)—phospholipid hybrid NPs (Scheme S1) have been developed to target human pancreatic cancer cell lines at different malignancies by actively augmenting NPs’ surface with specific epitopes, enhancing its uptake within the cell.^24,25^ Delivery of GBCAs through albumin-, adenosine-, or glutamine-coated polymeric NPs could provide higher MR contrast as means of higher cellular uptake.

Various immunoglobin G (IgG)-functionalized silver NPs were used to successfully target Pancreatic adenocarcinoma (PDAC) cells;^26–28^ however, our hybrid PLGA-lipid NPs uniquely exploit specific metabolic pathways that are highly active in PDAC, thus potentially offering enhanced selectivity and sensitivity for diagnostic imaging.

Albumin is the most abundant protein in the blood, and tumor cells internalize it through micropinocytosis from the extracellular matrix to produce growth supporting amino acids, such as glutamine and proline.^29–31^ Adenosine is a naturally occurring purine nucleoside, highly expressed in neoplastic microenvironments with pro- and antitumorigenic effects, depending on the receptors engaged on the different cell types.^32^ Pharmacological inhibition of G-protein-coupled adenosine receptors, A1a, A2a, A2b, and A3, in pancreatic cancer cell lines did not affect intracellular levels of adenosine; instead, blockade of equilibrative nucleoside transporters (ENTs) and concentrative nucleoside transporters (CNTs) by dipyridamole decreased their intracellular concentration, confirming their potential role in the uptake of adenosine-coated NPs via nucleoside transporters.^33^ While not essential to normal cells, glutamine becomes an essential amino acid for cancer cell survival and proliferation. The Warburg effect best describes their preference to increase glycolysis for energy production, even in the presence of oxygen.^29^ Inhibition of glutamine metabolism disrupts cancer cell growth and increases oxidative stress, highlighting the cancer cells’ reliance on glutamine.^34^

These concepts suggested that broadly targeting cancer metabolism could provide a therapeutic avenue for enhanced cellular uptake of surface-decorated Gd-loaded PLGA-NPs in pancreatic cancer, setting a steppingstone for future in vivo therapeutic applications.

## 2 Materials and Methods

### 2.1 Chemicals and Buffers

The 1,2-distearoyl-*sn*-glycero-3-phosphoethanolamine-*N*-[methoxy­(polyethylene glycol)-2000] (ammonium salt) 18:0 PEG2000 PE and 1,2-distearoyl-*sn*-glycero-3-phosphoethanolamine-*N*-[carboxy­(polyethylene glycol)-2000 NHS ester] (sodium salt) were purchased from Avanti Polar Lipids Inc., Croda International Plc group (Alabama, US). Poly­(d,l-lactide-*co*-glycolide) (PLGA) 50:50 (P2191), average molecular weight (Mw) 30,000–60,000 Da, and poly­(vinyl alcohol) (PVA), Mw 31,000–50,000 Da (98–99% hydrolyzed), albumin from bovine serum (BSA), *N*,*N*′-dimethylformamide (DMF), Fmoc-Gln­(Trt)–OH glutamine Reagent, Piperidine, *N*,*N*′-diisopropylcarbodiimide, Oxyma, Trifluoroacetic, triisopropylsilane, hepes, NaCl, chloroform, methanol, deuterated water, Thiazoly Blue Tetrazolium Bromide (MTT), and all other salts and solvents were purchased from Sigma-Aldrich (Massachusetts, US) and used without further purification. The amphiphilic Gd-DOTAMA (C_18_H_37_)­2 was synthesized and purified according to previously reported procedure.^35^

Human pancreatic cancer cell lines (MiaPaca2 and PANC1) were obtained from the American Type Culture Collection (ATCC, Virginia, US). Dulbecco’s Modified Eagle Medium (DMEM) high glucose, fetal bovine serum (FBS), penicillin and streptomycin, l-glutamine (l-Gln), and MycoAlert PLUS Mycoplasma Detection Kit were obtained from Lonza Sales AG-EuroClone S.p.A. (Milano, It). Dialysis buffer solution was made with Hepes (3.8 mM) and NaCl 150 mM. pH and osmolarity were corrected to 7.3 ± 0.1 and 280 ± 20 mOsm/L), respectively. pH was measured by using three-point calibration with standard solutions (pH = 4.0, 7.0, and 10.0). Osmolarity was controlled by using a Knauer K-7400S Semi-Micro Osmometer upon three-point calibration with standard solutions (0, 300, and 850 mOsm/L).

### 2.2 Preparation of PLGA-NPs

PLGA-NPs were obtained using the oil-in-water (o/w) emulsion solvent extraction method.^13^ A scheme of the preparation method is reported in the Supporting Information (Scheme S2). Briefly, phase 1 emulsion was prepared by dissolving 25 mg of PLGA, 3.2 mg of Gd-DOTAMA (C_18_H_37_)_2_, 1 mg of DSPE-PEG (2000) methoxy, and 1.2 mg of DPPE-PEG (2000)­NHS in 0.5 mL of chloroform. Organic phase 1 solution was added to phase 2, a 3% *w*/*v* PVA aqueous solution (3 mL), drop by drop, under controlled stirring. The 2 phases were then emulsified with a sonicator (Ika T-25 Ultra-Turrax Digital Homogenizer with an Ika Dispersing Tool S25N-18G, Cole Parmer, Germany) tip for 2 and a half minutes at 100% power. The final emulsion was transferred to a 50 mL round-bottomed flask and attached to a rotary evaporator at 740 mmHg and 30 rpm for 120 min to remove the organic solvent. Free Gd-DOTAMA (C_18_H_37_)_2_ traces were removed by dialysis (Mwco 14 000 Da) at 4 °C in HEPES-buffered saline (HBS) dialysis buffer overnight. PVA solvent excess was removed to concentrate the final solution through 5 cycles of vivaspin (Sartorius) (CO of 1 × 10^6^) centrifugation at 4500 rpm for 20 min. Control NPs, without ligand coating, were synthesized using the same synthetic protocol without activated NHS ester groups. NPs were stored in darkness at 4 °C for further analysis.

### 2.3 Conjugation of BSA, ADN, and Glut to PLGA-NPs

A solution of BSA, ADN, or Glut in HBS (10, 1, and 1 mg/mL, respectively) was added to PLGA-NP solution after NP preparation using an NHS to ligand molar ratio of 1:5.

Synthesis of polyglutamine [6 glutamine residues, poly­(Q6)] was carried out by solid phase peptide synthesis (SPPS), as reported in Supporting Information and summarized in Scheme S3. For characterization, ^1^H NMR spectrum acquisition of polyglutamine was done using a Bruker 600 MHz (Supporting Information). BSA and ADN were purchased and used with or without further purification.

The conjugation reaction between the protein and PLGA-NPs was carried out at room temperature for 4 h under controlled stirring. The final reaction solution was centrifuged and concentrated via vivaspin (Sartorius) (CO of 1 × 10^6^) centrifugation cycles at 4500 rpm for 20 min. The observed relaxivity (*R*_1obs_) and amount of Gd-DOTAMA (C_18_H_37_)_2_ entrapped in PLGA-NPs were determined by ^1^H Nuclear Magnetic Resonance (nuclear magnetic relaxation dispersion (NMRD)) *T*1 measurement, at 21.5 MHz and 25 °C (Stelar Spinmaster, Mede, Italy), of the digested sample complex solution (6 mol/L HCL at 120 °C for 24 h). The Gd concentration was calculated using the following formula

$$\left[Gd\;mmol/L\right]=\left[(R_{1obs}-0.5)/13.7\right]\times 2$$

where *R*_1obs_ represents the observed longitudinal relaxation rate of the digested sample, 0.5 is the diamagnetic contribution of HCl, and 13.7 is the coefficient corresponding to the Gd relaxivity in 1 molar solution, all of which are multiplied by the dilution factor. The relaxivity was then normalized (*R*_1p_ mM^–1^ s^–1^) against the obtained Gd concentration, calculated as such

$$R_{1p}\;\left({mM}^{-1}\;s^{-1}\right)=\left(R_{1obs}-0.38\right)/\left[Gd\;mmol/L\right]$$

where *R*_1obs_ represents the measured longitudinal relaxation rate of the NP in an aqueous solution, 0.38 is the diamagnetic contribution of water, and [Gd mmol/L] is the metal concentration. Relaxivity of nanosystems is a measure of the efficiency of the Gd ions in enhancing the relaxation rate of water protons. The encapsulation efficiency (EE %) of Gd-DOTAMA (C_18_H_37_)_2_ was calculated with the following formula

$$EE\;Gd‐DOTAMA\;\%={Gd‐DOTAMA}_{encap}{/Gd‐DOTAMA}_{tot}\times 100$$

Where Gd-DOTAMA_encap_ is the amount of the encapsulated Gd complex and Gd-DOTAM*A*_tot_ is the amount initially added for PLGA synthesis. The hydrodynamic mean diameter of PLGA-NPs was determined using a dynamic light scattering (DLS) Malvern Zetasizer 3000HS instrument (Malvern, UK). All samples were analyzed at 25 °C in filtered HBS buffer (pH 7.4, filter CO 200 nm). The amounts of the conjugated ligands were determined using the following assays: BSA Bradford assay quantification was used to determine the protein concentration ligated from the final synthesis volume of the BSA-PLGA. Its CE %, was evaluated with the following formula

$$BSA‐CE\%=\left({BSA}_{ligated}/{BSA}_{tot}\right)\times 100$$

where BSA_ligated_ is the protein amount resulting from the Bradford assay and BS*A*_tot_ is the added protein from the conjugation reaction. Free ADN was quantified by means of the sulfur-penerol method from the resulting waste volume of the NPs vivaspin centrifugation. The CE % of linked ADN was then calculated by using the following formula

$$\text{ADN}‐CE\%=\left({ADN}_{tot}-{ADN}_{free}{/ADN}_{tot}\right)\times 100$$

where ADN_tot_ is the total adenosine used for the ligation and ADN_free_ is the excess adenosine washed away from the vivaspins. Free Polyglutamine was quantified by means of BCA assay from the resultant vivaspin excess volume.

The CE % of Polyglutamine was calculated with the following

$$Glut‐CE\%=\left({Glut}_{tot}-{Glut}_{free}{/Glut}_{tot}\right)\times 100$$

where Glut_tot_ is the total glutamine used for the initial ligation reaction and Glut_free_ is the glutamine in the excess volume washed away from the vivaspins. The average number of BSA, ADN, and Glut molecules conjugated to PLGA-NPs was calculated by dividing the number of ligand molecules found in the solution for the calculated average number (*n*) of PLGA-NPs. The average number (*n*) of PLGA-NPs was calculated using the calculator tool in the DLS instrument. Resulting NPs were kept under dark at 4 °C for further analysis.

### 2.4 Relaxometric Quantification of Metal Concentration

The concentration of Gd­(III) in the NPs was determined by using a ^1^H relaxometric approach. Briefly, 70 μL of specimens was added to an equal volume of 37% HCl and placed into sealed glass vials overnight at 120 °C. Then, the water proton 1/*T*_1_ (=*R*_1_) longitudinal relaxation rate of acidic solutions was measured at 25 °C by using a Stelar SpinMaster relaxometer and the concentration of metal was calculated based on previously obtained calibration curves of Gd (III) aqua-ion solutions.

### 2.5 Proton Nuclear Magnetic Relaxation Dispersion 1/*T*_1_ Profiles

Measurements were carried out at 25 °C over a range of magnetic field strengths from 0.00024 to 0.47 T (corresponding to 0.01–20 MHz proton Larmor Frequency) on a Stelar field-cycling relaxometer (Stelar, Mede, Italy), under complete computer control with an absolute uncertainty of 1%. Data points from 0.5 T (21.5 MHz) to 1.7 T (70 MHz) were collected on a Spinmaster spectrometer (Stelar, Mede, Italy) working at variable magnetic fields.

### 2.6 PLGA-NPs’ Size and *R*_1obs_ Stabilities

To perform size and *R*_1obs_ stability tests, PLGA, BSA-PLGA, ADN-PLGA, and Glut-PLGA NPs were diluted in HBS buffer or human serum (Sernorum) (Sigma-Aldrich) and left at 37 °C, under continued stirring for 7 days. The *R*_1obs_, size, and ζ-potential of each PLGA-NP sample were monitored on the Stelar field-cycling relaxometer operating at 21.5 MHz and DLS, respectively, both at 25 °C.

### 2.7 Cell Lines

MiaPaca2 and Panc1 cells were cultured in DMEM high glucose medium supplemented with 10% (v/v) FBS, 100 U/mL penicillin, 100 U/mL streptomycin, and 2 mM l-glutamine. Cells were routinely passed twice a week. Briefly, cells were gently washed with sterile phosphate-buffered saline (PBS) to remove the residual media. Then, cells were detached with 0.25% trypsin–EDTA solution for 3–5 min at 37 °C until cells were fully dissociated. Fresh medium was added to neutralize trypsin activity, and cell suspension was centrifuged at 1100 rpm for 5 min. The supernatant was finally discarded, and the cell pellet was resuspended in a fresh medium. Cells were then seeded into a new culture flask and incubated at 37 °C under a humidified atmosphere of 5% CO_2_. Cells were negative for mycoplasma as tested by using a MycoAlert Mycoplasma Detection Kit (Lonza Sales AG-EuroClone S.p.A., Milano, It).

#### 2.7.1 In Vitro Cell Viability

The MTT test was used to evaluate the in vitro cytotoxicity of the PLGA-NPs on MiaPaca2 and Panc1. Both cell lines were seeded at a density of 1 × 10^4^ viable cells/well in 96-well plates for 24 h before experiments. Cells were incubated with PLGA-NPs for 24 h at increasing concentrations of Gd ranging from 0 to 80 μM. The medium was then discarded from the wells and washed with 100 μL PBS. From a stock solution of MTT (5 mg/mL) in PBS, an aliquot of 10 μL was mixed with 100 μL of DMEM medium for each seeded well. After 4 h of incubation, the culture medium with MTT solution was discarded and crystals of formazan dissolved in 150 μL of dimethyl sulfoxide. The absorbance of the resulting coloration was read on a microplate reader (GloMax Microplate Reader, Promega) at a 560 nm wavelength. Cell viability was calculated as a fraction of viability against the controls.

#### 2.7.2 Cellular Uptake Experiments

For in vitro uptake experiments, around 7 × 10^5^ MiaPaca2 or Panc1 were seeded in 6 cm diameter Petri dishes and left for 24 h to proliferate. Then, cells were incubated for 24 h at 37 °C in the presence of PLGA, BSA-PLGA, ADN-PLGA, or Glut-PLGA NPs at 80, 50, 30, 20, and 10 μM Gd concentrations. At the end of the incubation, cells were washed three times with 5 mL of ice-cold PBS, detached with trypsin/EDTA, and transferred to falcon tubes. Cell samples were sonicated at 30% power for 30 s in ice, and their protein concentrations were determined by a Bradford assay (Biorad, Hercules, CA, USA). Sample digestion was performed with concentrated HNO_3_ (70%, 1 mL) under microwave heating (Milestone MicroSYNTH Microwave labstation). The Gd content in the cell samples was determined using inductively coupled plasma (element-2; Thermo-Finnigan, Rodano (MI), Italy).

### 2.8 MRI

All MRI images were acquired on a Bruker Avance 300 spectrometer (7T) equipped with a Micro 2.5 microimaging probe (Bruker BioSpin, Ettlingen, Germany). Following the final incubation step in the uptake experiments, MiaPaca2 and Panc1 cells were washed, detached, centrifuged, and resuspended in 0.07 mL of PBS. The suspensions were aliquoted in glass capillaries containing cell-retained Gd from prior incubation (10–80 μM), centrifuged at 1100 rpm for 5 min to form cell pellets, and fixed in a conical glass tube containing water. The phantom was imaged using a standard *T*_1_-weighted multislice spin–echo sequence (TR = 200 ms; TE = 3 ms; NEX = 6; FOV = 15 × 15; MTX = 128 × 128) and T1 map (TR = 6000, 3000, 1500, 800, 600, 400, 200, 100, 50 ms; TE = 3 ms; FOV = 5 × 5; MTX = 128 × 128). The T1 relaxation times were calculated by using a standard saturation recovery spin echo. The *T*_1_values were obtained from the *T*_1_map scans by manually drawing in the ROI, within the circumference of the glass capillaries. The longitudinal water proton relaxation rate (1/*T*_1_) of each sample was then calculated and expressed as a percentage. The cell pellets were then recovered from the capillaries with an elongated glass pipet and suspended in 0.2 mL of PBS. The recovered samples were stored for further sonication, protein assay, and sample digestion.

### 2.9 Statistical Analysis

Data were calculated from at least 3 independent experiments and expressed as means ± standard error of the mean or ± SD (standard deviation of the mean), as indicated in the different determinations. Statistical analyses were performed using the student multiple *t*-test and One-way ANOVA. A p-value less than 0.05 was considered statistically significant.

## 3 Results and Discussion

### 3.1 Preparation of PLGA NPs

PLGA NPs are one of the most effective biodegradable delivery platforms in cancer nanomedicine.^36^ To obtain them, the *o*/*w* emulsion solvent extraction method was used^37^ (Scheme S1). Briefly, PLGA (P2191) 50:50, DSPE-PEG(2000)­methoxy, DPPE-PEG(2000)­NHS, and Gd-DOTAMA (C_18_H_37_)_2_ were dissolved in chloroform. The organic phase was added drop by drop into an aqueous phase of poly­(vinyl alcohol) surfactant (PVA), allowing the polymer to nucleate. Choosing the surfactant concentration is important to facilitate the emulsion and obtain the desired particle size.

An excess of PVA would decrease the final size, while an insufficient amount would fail NPs’ stabilization, leading to aggregation and precipitation.^38^ The fate of the NPs upon administration is strictly determined by its surface properties and size.^39,40^ In vivo studies showed that small particles of less than 30 nm are eliminated by renal filtration and larger ones, 150 to 300 nm, are phagocytised by the mononuclear phagocytic system cells mainly present in the liver and the spleen.^39,40^ Our aim was to produce particles about 150 nm in diameter; hence, PVA was kept at 3%, yielding uniform, small sized and monodispersed NPs,^41^ as also shown in previous reports.^13,25^ Furthermore, NPs with a hydrodynamic diameter between 100 and 150 nm have previously been used for passive tumor targeting due to the EPR effect. During tumor development, cancer cells secrete VEGF and growth factors to stimulate angiogenesis, supporting the formation of blood vessels that provide the nutritional and oxygen supply for growth. These structures are usually abnormal in form and structure characterized by discontinuous endothelium, leaving wide fenestrations of 200 to 800 nm and allowing particles to passively accumulate in the tumor.^42–44^

### 3.2 UPLC and Mass Analysis of Synthesized Polyglutamine

The targeting route exploited herein is based on the administration of NPs externally decorated with polyglutamine (Glut). For this purpose, the synthesis of Glut was carried out successfully by SPPS on Rink Amide ProTide Resin, using a Liberty Blue automated peptide synthesizer (Scheme S2, Supporting Information).

After cleavage from the resin, polyglutamine was checked by analytical UPLC, by means of an ACQUITY UPLC system equipped with both UV–vis and MS detectors. A HSS T3 column (1.8 μm, 2.1 × 100 mm) was used with gradient elution (solvent A: 0.05% trifluoroacetyl in H_2_O; solvent B: CH3CN) from 0% to 5% in 6 min and from 5% to 80% in 1 min at a flow rate of 0.4 mL min^–1^). Polyglutamine was characterized by a UPLC Acquity H-Class coupled with the QDa mass detector and TUV detectors. The chromatogram displays a single, prominent peak with a retention time of 2.07 (Figure 1A). This dominant component (tR 2.07 min, λ = 220 nm) has a purity of ≥85%. Minor peaks observed at earlier retention times correspond to synthesis of byproducts or shorter polyglutamine chains. The mass spectrometric analysis (Figure 1B) revealed two significant signals, providing insight into the peptide’s protonation states. The first signal, ESI + MS *m*/*z* (calcd For C_30_H_51_N_13_O_12_, MW 785.82): [M + 2H]^2+^ 393.44 (obsd), 393.91 (calcd), corresponds to the doubly protonated form of the polyglutamine peptide. The second signal, [M + 1H]^1+^ 785.88 (obsd) and 786.82 (calcd) corresponds to the singly protonated form of the molecule.

**Figure 1:**
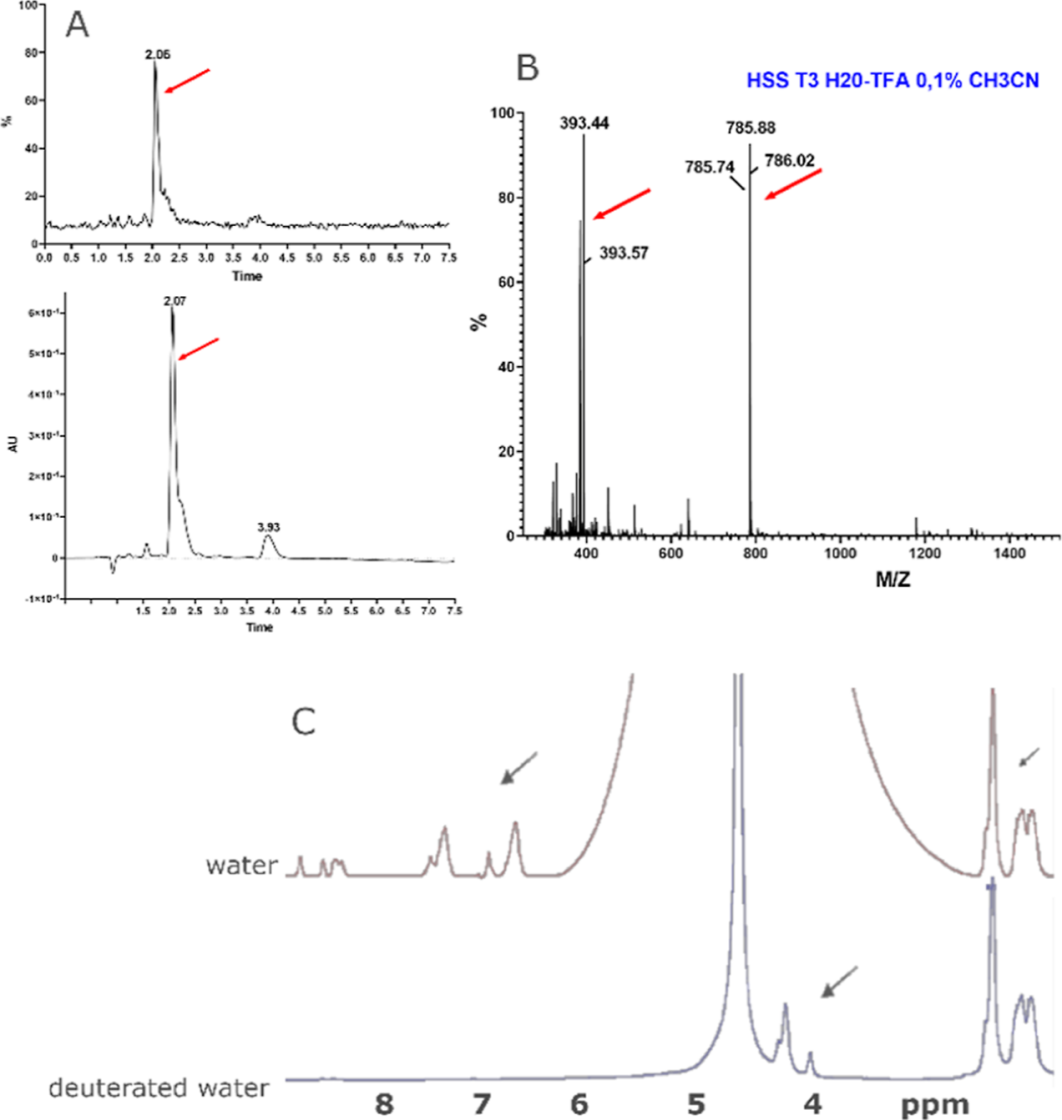
(A) Extracted UPLC chromatogram of Glut. (B) Mass spectra of synthesized Glut. (C) ^1^H NMR spectrum of synthesized Glut in H_2_0 and D_2_0 at 298 K.

The consistency between the theoretical and observed mass-to-charge ratios supports the structural integrity and purity of the synthesized polyglutamine. These data provide validation for the synthesis process, ensuring that the polyglutamine peptide is suitable for further experimental research.

### 3.3 NMR Spectra of Poly-Glutamine

In this study, two ^1^H NMR spectra were obtained for synthesized polyglutamine using H_2_O and D_2_O as solvents, respectively, revealing significant insights into the molecular characteristics and solvent interactions of the sample. The H_2_O spectrum exhibited a broad water proton peak between 3 and 6 ppm, a common feature due to high proton concentration, which overshadowed other signals. Notably, exchangeable protons from amide groups were present, manifesting between 6.0 to 8.5 ppm, consistent with the literature that highlights these regions for such functional groups in polypeptides.^45,46^ The three peaks around 2.4 to 2.6 ppm, well detectable using D_2_O as solvent, validated the glutamine structure (Figure 1C).^47^ Exchangeable peaks between 6.0 to 8.5 ppm are not detectable using D_2_O as solvent due to proton exchange with deuterium.

### 3.4 BSA, ADN, and Glut Surface Conjugation to PLGA-NPs

Prior to surface conjugation, NPs underwent a dialysis washing step against HEPES Buffered Salt (HBS) to remove unbound Gd­(III) chelates and then were centrifuged to separate aggregates and further impurities. The supernatant was collected and washed from excess PVA with vivaspin centrifugation cycles. Removing excess surfactant from the solution allows for higher surface-to-ligand contact between the activated NHS esters on the surface of the NPs and the reactive groups of the ligand, ensuring that most of the particle reactive sites bind to the amine groups of the latter. All formulations demonstrated size ranges considered to be suitable for potential (intravenous) *i.v.* administration and prolonged-circulating half-life properties. In Table S1 and Figure 2, particle size is reported by considering the hydrodynamic diameter obtained from DLS, with standard deviations calculated from three independent batches prepared on separate days. The resulting diameter measurements correspond to 140 ± 6, 155 ± 5, 146 ± 11, and 149 ± 15 nm for PLGA, BSA-PLGA, ADN-PLGA, and Glut-PLGA, respectively. Their polydispersity index (PDI) was kept below 0.2 and exhibited a proportional pattern in relation to their size. The ζ-potential reflects the average surface charge distribution, with values suggesting a neutral to slightly negative surfaces, consistent with PEGylated NPs.^48^ The mean particle ζ-potentials were approximately −4. ± 1.6, −4.5 ± 2, - 3.0 ± 0.6, and −2.9 ± 0.6 mV for PLGA, BSA-PLGA, ADN-PLGA, and Glut-PLGA, respectively (Table S1 and Figure 2).

**Table 1:** Obtained Parameters from the ^1^H NMRD Profile Analysis of the Formulated PLGAs

	PLGA/Gd	PLGA/Gd/ADN	PLGA/Gd/Glut	PLGA/Gd/BSA
τ_RL_/ns	1.2 ± 0.1	1.2 ± 0.1	1.3 ± 0.1	3.3 ± 0.3
τ_RG_/ns	80^a^	80^a^	80^a^	80^a^
*S* ^2^	0.41 ± 0.02	0.41 ± 0.02	0.42 ± 0.01	0.45 ± 0.02
τ_M_/ns	640 ± 20	700 ± 35	600 ± 22	540 ± 30
*q*	1^a^	1^a^	1^a^	1^a^
*r*/Å	3.0^a^	3.0^a^	3.0^a^	3.0^a^
*a*/Å	3.8^a^	3.8^a^	3.8^a^	3.8^a^
^298^*D*/10^–10^ m^2^ s^–1^	2.24^a^	2.24^a^	2.24^a^	2.24^a^

a Parameters were fixed during fitting analysis.

**Figure 2:**
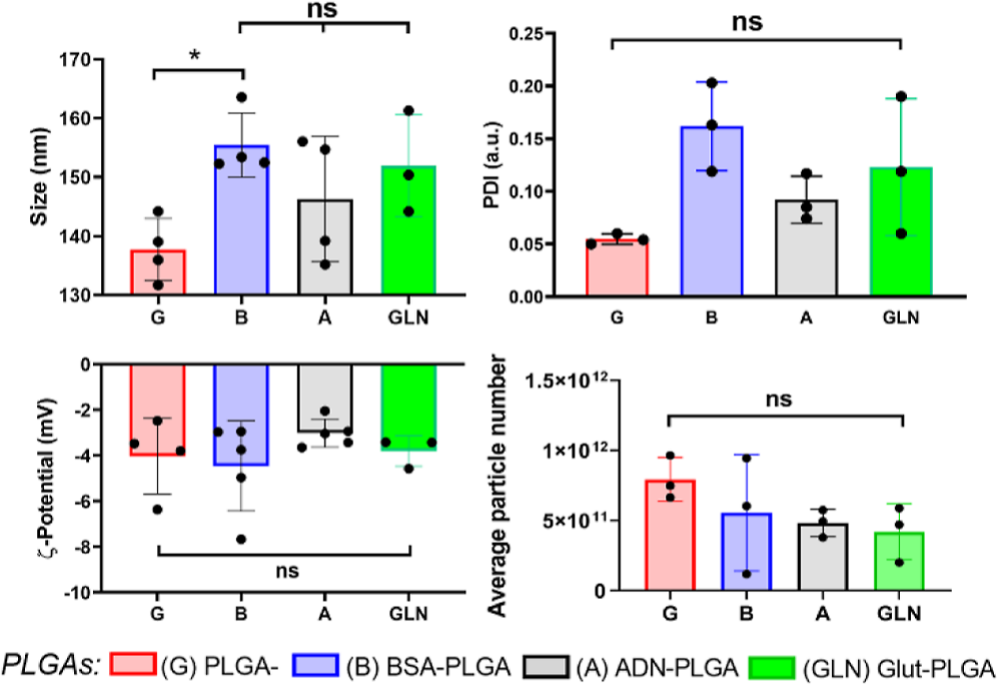
Physiochemical characterization of polymeric NPs such as size, PDI, ζ-potential, and average NP number from the formulated PLGAs were assessed. NPs from the BSA targeted group displayed significant size increase compared to the untargeted group (B vs G). PDI was kept below 0.2, indicating monodispersed particle population. NPs’ ζ-potential remained unaltered after ligand conjugation in all groups (*n* = 3 independent NP batches per group). Results are shown as mean ± SD. The Mann–Whitney test was used to determine statistical differences between the groups, with *p < 0.05.

Surface charge and PDI were similar between the groups. BSA-PLGA experienced an increment in size of around 15 nm compared to the preconjugation one, because of the surface binding of a high molecular weight molecule. The resulting surface charge was mostly attributed to the characteristics of the hydrophilic pegylated shell and the corresponding ligand. BSA is an anionic protein at physiological pH and adsorption onto NPs’ surface may impart a negative charge to the NPs.

Although not significant, BSA did indeed decrease the NP surface charge after conjugation. Similarly, ADN and Glut conjugation slightly increased the surface charge of NPs, though unaltering its negative charge. The negative ζ-potentials obtained are less favorable for cellular uptake due to the negatively charged nature of cellular membranes. Despite this, for in vivo applications, a slightly negative charge prolongs NPs’ blood circulation time, which accordingly enhances biodistribution inside the tumor.^49^

Longitudinal relaxivity values (*r*_1p_) for PLGA, BSA-PLGA, ADN-PLGA, and Glut-PLGA were calculated to be 23.9 ± 1.9, 27.0 ± 2.8, 22.1 ± 1.6, and 23.2 ± 2.4 m^–1^ s^–1^, respectively, at 21.5 MHz and 298 K (Table 1). All the formulated PLGAs demonstrated a good Gd (III) complex encapsulation efficiency (EE %) of above 80%. To determine the grade of functionalization for each group, the ratio of ligand molecules attached to the surface of each PLGA was calculated, resulting in 3.5 × 10^6^, 2.6 × 10^6^, and 5.2 × 10^6^ ligand molecules/NP for BSA, ADN, and Glut, respectively. It corresponds to a conjugation efficiency (CE %), in percentage, of 77%, 65%, and 99%.

### 3.5 Proton Nuclear Magnetic Relaxation Dispersion 1/*T*_1_ Profiles

The relaxivity values for the different formulations were measured as a function of the applied magnetic field at 298 K, thereby obtaining the NMRD profiles. A mathematical analysis of these curves is essential for extrapolating the molecular and dynamic parameters responsible for the relaxivity values. The ^1^H NMRD of the different samples exhibits a shape characteristic of systems, in which Gd­(III) complexes undergo slow rotation, with a peak at approximately 30 MHz (Figure 3).

**Figure 3:**
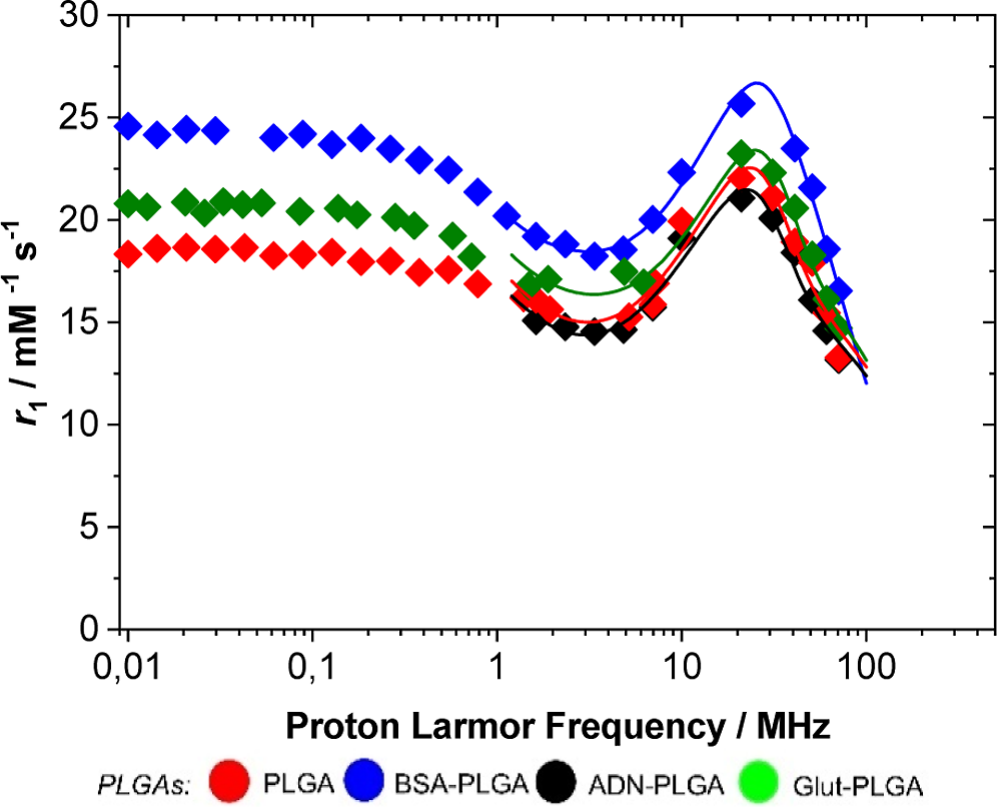
Profiles of 1/*T*_1_^1^H-NMRD (pH 7.4, 25 °C) of PLGA-NPs. NMRD analysis was conducted using the parameters reported in Table 1.

As a general comment, the restriction of mobility of the confined Gd­(III) chelates is promoted by the strong interactions between the steric lipophilic chains of the complex molecules and the hydrophobic PLGA polymer chains. This observation strongly reinforces the potential of PLGA NPs encapsulated with amphiphilic Gd complexes compared to hydrophilic ones for imaging applications. The rotational dynamics were analyzed using the Lipari-Szabo model-free approach, which describes both local (τ_RL_) and global (τ_RG_) rotational correlation times through an order parameter, *S* ^2^.

If the reorientational motion of the Gd­(III) complex is independent of the global tumbling of the NPs, then *S* ^2^ = 0. Conversely, if the complex is immobilized on the surface of the NPs, *S* ^2^ = 1. The *r*_1p_ value of BSA-PLGAs was approximately 17% higher with respect to the unconjugated NPs at 30 MHz. This increase is primarily attributed to an increase in τ_RL_ from 1.2 ns for PLGA/Gd to 3.3 ns for PLGA/Gd/BSA. A slight reduction in the water residence lifetime was also observed, although this parameter is not accurately extrapolated from this analysis. However, the values show a similar order of magnitude to those calculated for other nanosystems functionalized with Gd-DOTAMA derivatives (see Botta and Aime’s articles). During fitting, τ_RG_ was fixed at 80 ns, consistent with literature (same references as above), with *D* (2.24 × 10^–10^ m^2^ s^–1^), rGd–H (3.0 Å), and a Gd–H (3.8 Å) (Table 1).

### 3.6 Assessment of Stability by Size and Relaxivity

Stability of the PLGAs’ formulations was assessed for 4 days by measuring their size, ζ-potential, and the relaxivity using DLS and the SpinMaster instrument operating at 21 MHz, respectively. Figure 4A–C shows that all NPs are stable in size in both Hepes Buffered Salt (HBS) and human serum. Additionally, the ζ-potential of each PLGA-NP sample in HBS did not change in the analyzed time (Figure S1).

**Figure 4:**
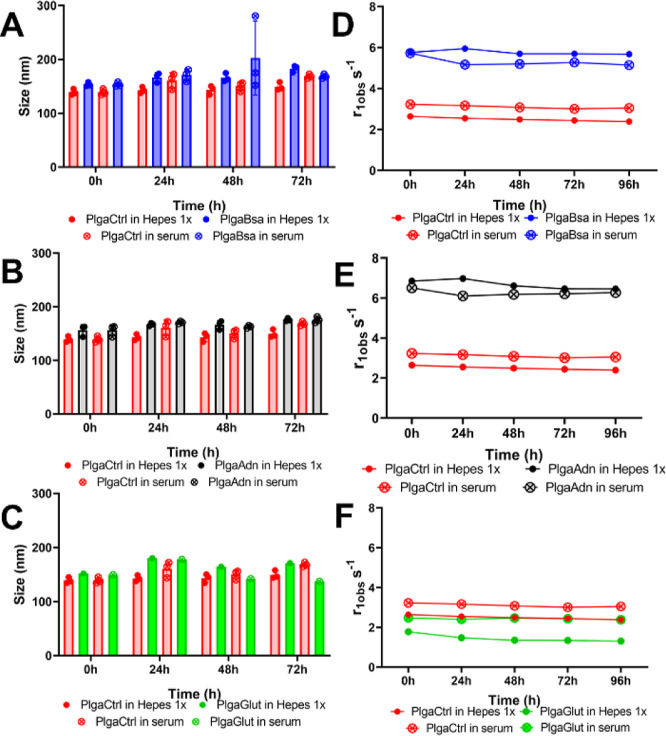
Targeted PLGA-NPs’ size stabilities were measured by DLS, at 25 °C, over a course of 3 days in HBS and human serum and compared to control PLGA group (A–C). Gd complex stability was evaluated by measuring the r1obs in a Stelar Spinmaster at 21.5 MHz over the course of 4 days (D–F).

This suggests that human serum has little affinity toward these amphiphilic Gd-DOTAMA (C_18_H_37_)_2_ complexes, indicating that their linkage to the PLGA-NPs’ surface will be stable when administered *i.v.* control PLGA NPs were more stable as their size underwent little change after incubation with HBS. All targeted PLGAs were subject to a slight size increase going from around 150 to 180 nm after incubation with HBS. In human serum, targeted PLGAs behaved similarly to its analogue HBS profile. The control PLGA increased in size after 72 h of incubation, possibly attributed to slow unspecific adsorption from the serum to the free available surface of the particle. Analogously, measurement of relaxivity did not change during the measurement time, indicating a structural rigidity of the NPs and capacity at holding the Gd complex within its structure without altering its properties (Figure 4D–F).

### 3.7 In Vitro Cell Viability

Biocompatibility of NPs was tested in vitro by (i) hemolysis tests on red blood cells and (ii) viability assays performed on pancreatic MiaPaca2 and Panc1 cells.

The first test was used to assess the eventual interaction of NPs with biological cell membranes. RBCs were used for this purpose since they are the most abundant cells in the blood, so their interaction with NPs has to be tested in view of possible future *i.v.* administration. Data showed the absence of RBC lysis upon incubation with all four formulations (30 min, concentration of 80 μM, Figure S2).

The second test on cell culture was carried out to confirm the biocompatible nature of the polymer and establish an ideal dose with the lowest toxicity for cellular uptake experiments. Each cell line was incubated with PLGA, BSA-PLGA, ADN-PLGA, and Glut-PLGA at increasing μM concentrations for 24 h (Figure 5).

**Figure 5:**
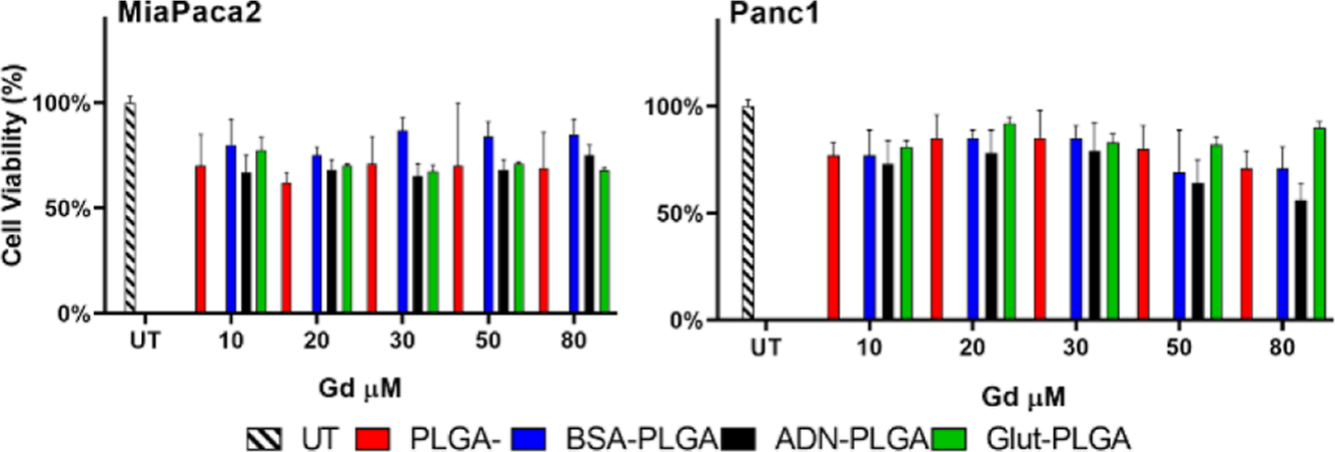
Percentage of viability (measured with MTT assay) of MiaPaca2 and Panc1 cells after 24 h of incubation at increasing concentrations of Gd-loaded PLGAs. Graphs show the mean ± SD of percentage of viability evaluated on three independent experiments.

The highest Gd concentration used for the MTT assay corresponds to the minimum dose required to achieve a detectable MR signal. Cell viability levels were evaluated against ISO 10993-5 standards for in vitro cytotoxicity, which recommend viability thresholds above 70% for biocompatibility.^50^ The low toxicity of the control PLGA group demonstrated the biocompatibility of these “*naked*” nanosystems. Cells may incorporate a higher concentration of extracellular components depending on their metabolic needs. Applying an essential metabolite, or a byproduct of it, to NPs’ surface may induce a higher internalization and release of the Gd complex thus increasing toxicity. In fact, ligation of BSA, ADN, or Glut faintly decreased cell viability, although at acceptable levels. The observed increase in toxicity in the BSA-PLGA group may account for a higher PLGA uptake mediated by the high KRAS-induced macropinocytosis activity characteristic of pancreatic cancer cells. The toxicity due to ADN may be attributed to increased uptake mediated by ENTs and CNTs receptors. In the Glut group, toxicity may be attributed to receptor-mediated internalization of solute carrier family 1, member 5 (SLC1A5). The viability, in percentage, of MiaPaca2 after 24 h incubation at 80 μM Gd with PLGA, BSA-PLGA, ADN-PLGA, and Glut-PLGA were 70%, 85%, 75%, and 70% and 71%, 71%, 56%, and 90% for Panc1, respectively.

### 3.8 Cellular Uptake

A preliminary assessment about internalization of NPs inside RBCs was carried out to exclude the specific loading of NPs eventually occurring after in vivo intravascular administration of NPs. RBCs were placed ex vivo in the presence of the four formulations of NPs for 3 h (concentration of 80 μM). Later, they were extensively washed in buffer and recollected by centrifugation. Cells content was quantified by using the Bradford assay and the internalization of Gd-containing-NPs by quantification of the ICP–MS measure of Gd.

The comparison of the cellular uptake efficiencies and the resulting contrast enhancement of the formulated Gd-loaded PLGA-NPs were assessed by acquiring MR images at 7T (Figure 6A).

**Figure 6:**
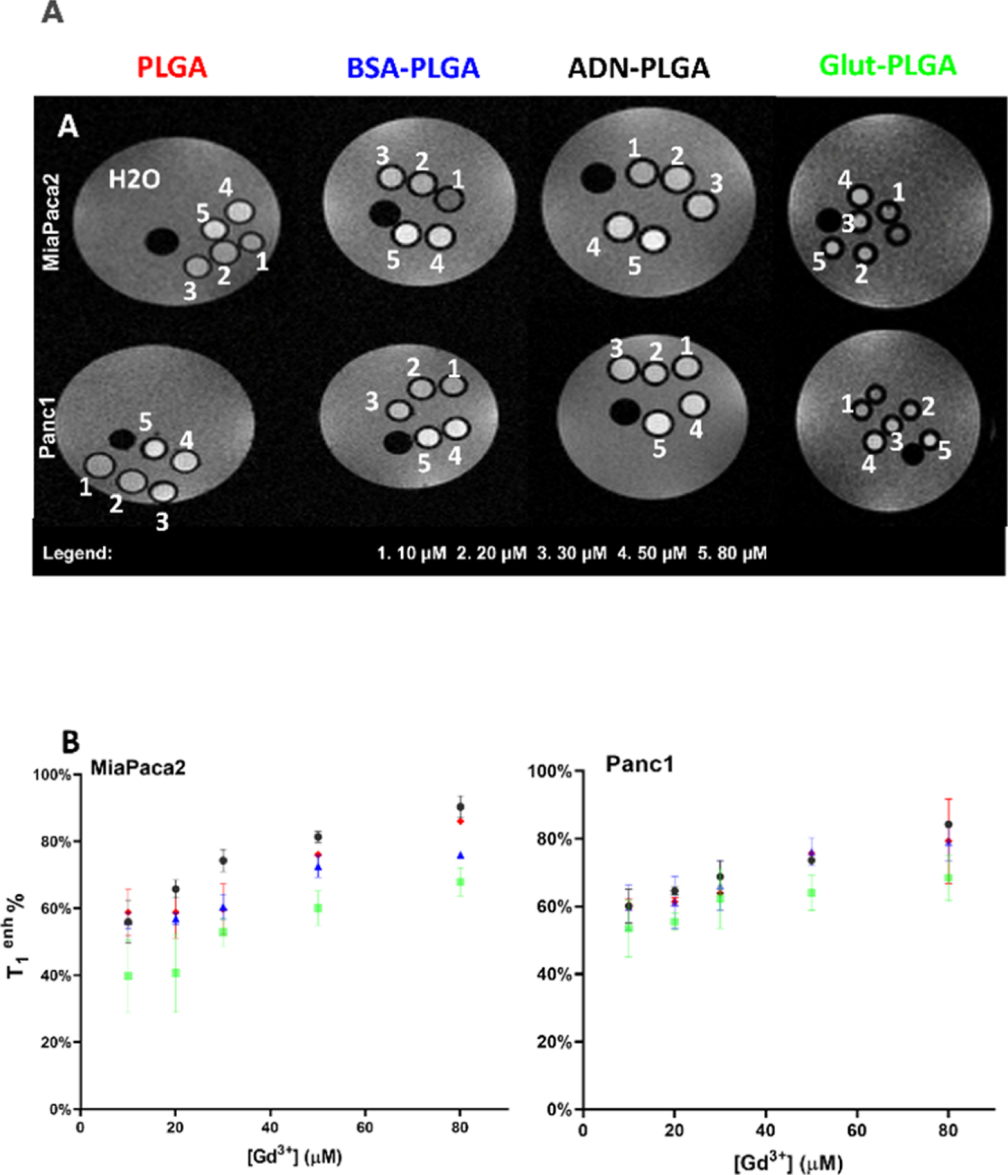
(A) 7T MR images of *T*_1_-weighted 5 mm glass tubes containing MiaPaca2 or Panc1 cells incubated with PLGAs at variable concentrations of Gd. (B) Comparison of *T*_1_ enhancements between PLGAs at 7T MRI.

As a result, no Gd internalization was assessed for all four tested NP formulations, so the aspecific uptake was excluded from RBCs (data not shown).

After 24 h of incubation at varying concentrations of Gd, the cells were collected and pelleted into 5 mm diameter glass capillaries. Signal enhancement was obtained from *T*_1_map 7T MR images. The measured signal intensity of the Gd-DOTAMA (C_18_H_37_)_2_ within the capillaries was able to increase sensitivity even at the lowest concentration, which is a primary imaging probe requirement to be visualized and detected by the instrument (Figure 6B).

It is worth noting that by increasing the concentration of Gd-bearing-NPs in the incubation medium, there is an increase in the *T*_1_^enh %^ in MR images of cells pellets (Figure 6B), as consequence of a larger amount of Gd complexes internalized inside cells (in endosomes, through micropinocytosis, see following Figure 7 for quantification). However, this increase is linear only at a low concentration. By increasing the amount of loaded Gd complexes, there is a saturation in the *T*_1_^enh %^ signal. This “quenching” of relaxivity has been largely analyzed in the literature, and it takes place when the difference in the relaxation rates between endosomes and cytoplasm is higher than the exchange rate (*k*_ex_) between the two compartments, that is, *IR*_1_^intra^ – *R*_1_^extra^ *I* > or ≫ *k*_ex_.^51^ Together with the *T*_1_-“quenching”, the high payload of paramagnetic species in the endosomes causes a dramatic decrease of *T*_2_* and both the conditions hamper the enhance of signal in *T*_1w_-MR images (Figure 6A,B).

**Figure 7:**
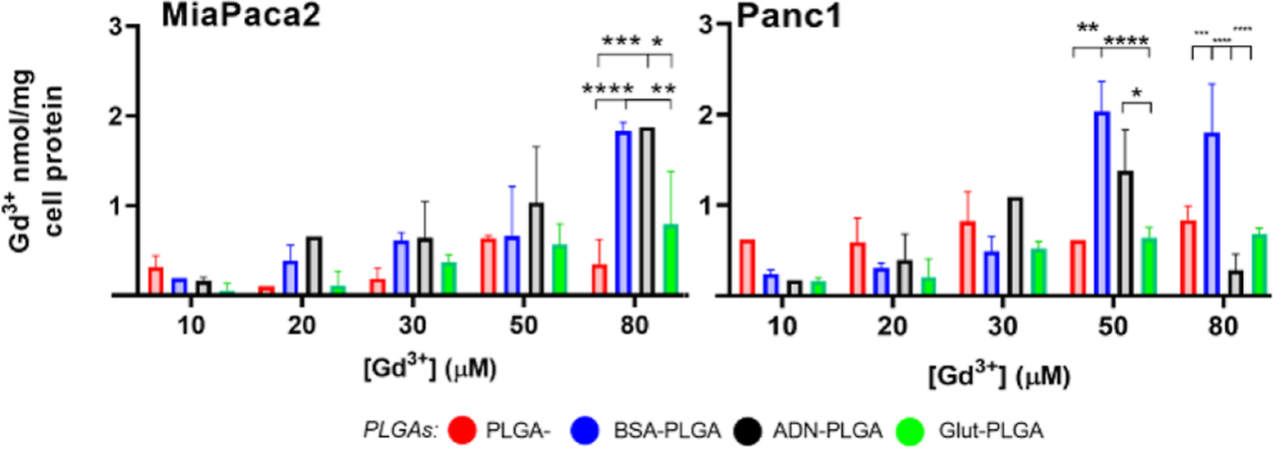
ICP–MS quantification of Gd retained intracellularly by MiaPaca2 and Panc1 cells after 24 h incubation. Graphs show the mean ± SD of internalized Gd nmol per mg of total cell proteins from 3 independent experiments. (***p < 0.01, **p < 0.1, *p < 0.5).

Cell pellets were then recollected for protein quantification and sample digestion to measure the Gd concentration via ICP–MS and finally determine the nmol Gd/mg cell protein ratio (Figure 7). This result allows for the quantification of PLGA NPs internalized inside cells.

Surface-treated NPs with BSA and ADN showed high uptake in both cell lines at greater concentrations, 50 and 80 μM, confirming cells’ preference to endocytose albumin for glutamine production and adenosine through the dynamic exchange between the extracellular and intracellular spaces. In the ADN-PLGA group at 80 μM, Panc1 showed substantial decreased uptake of NPs compared to both BSA and untargeted groups. The mechanism of adenosine internalization is concentration-dependent; therefore, adding a higher amount of ADN-carrying particles in the extracellular space might hamper this mechanism, resulting in poor intracellular delivery of the contrast agent.

Glut-PLGA showed the lowest uptake among PLGA formulations at all of the concentrations. The l-Glutamine constituent of the culture medium may have hindered the intracellular uptake of Glut-PLGA, although preliminary experiments did not point in this direction (data not shown). Further studies will be needed to evaluate the possible role of glutamine present in the cell culture medium.

Finally, to gain further insights into the internalization of NPs, preliminary experiments were conducted by using confocal microscopy for the detection of carboxyfluorescein-labeled-PLGA-NPs either *w*/*o* surface modification or upon coating with BSA. Data, reported in Figures S3 and S4, show that in both cell lines at 30 min or 24 h of incubation, the fluorescence signal is cytoplasmatic, thus confirming a rapid uptake of the system and endosomal escape after loading.

## 4 Conclusions

This study instigates the use of targeted NPs for imaging PDAC cells. In comparison to the existing functionalized NPs and imaging methods for PDAC, our study introduces a novel approach by leveraging hybrid PLGA-lipid NPs functionalized with metabolic ligands, such as albumin, adenosine, and glutamine.

Two considerations have to be taken into account for comparing the NPs reported herein to previously reported ones, i.e., (i) the kind of NP and (ii) the targeting agent attached to NP surface.

Concerning the chemical nature of NPs, previous research has explored a variety of formulations, including polymeric, inorganic, and liposomal systems, to enhance MRI contrast in PDAC detection.^52^ Polymeric NPs, such as PLGA-based systems, have demonstrated high biocompatibility easiness of preparation and controlled drug release, being considered a well-suitable system for theranostic applications.^53^ Inorganic NPs, including SPIONs and gold nanostructures, have been also largely investigated, especially because they offer higher MRI contrast (due to their magnetic properties).^54^ However, their application is strongly hampered by non-optimal biocompatibility.

Also, liposomal NPs have also been investigated for their ability to encapsulate imaging agents/drugs and facilitate prolonged circulation, as well as for improved biocompatibility.^55^ Both liposomes and PLGA-NPs represent good possibilities for targeted imaging and/or therapy, and the choice of one or the other systems is strongly linked to the nature of drugs to be delivered, if mainly hydrophilic or hydrophobic.

The hybrid PLGA-lipid NPs used herein offer a distinctive advantage by combining a biodegradable polymeric core with a lipid shell to improve stability, biocompatibility, and tunable surface functionalization, thus mixing some advantages of PLGA NPs and of liposomes. The presence of pegylated phospholipids can be strongly advantageous for both improving blood lifetime and making bioconjugation with targeting ligands easy (using the NHS strategy).

The second point deals with the targeting agent attached to the surface of the NPs. The functionalization with metabolic ligands provides an innovative targeting strategy that enhances NP uptake through active metabolic pathways rather than solely relying on passive diffusion (EPR effect), which is significantly hindered in PDAC due to its dense stromal microenvironment and poor vascularization.

Albumin, for instance, is abundantly internalized by PDAC cells through micropinocytosis to support amino acid metabolism, making it a promising targeting agent. Similarly, adenosine uptake is regulated by nucleoside transporters, which are overexpressed in the tumor microenvironment, and glutamine metabolism plays a crucial role in cancer cell survival, providing an additional route for targeted NP delivery. Hence, the metabolic targeting strategy exploits fundamental cancer cell processes, allowing for enhanced specificity and higher imaging sensitivity.

This can overcome immunogenicity and limited penetration in tumor tissues, which are linked to the use of antibody conjugation for active targeting, as commonly reported for SPIONs and gold-based NPs.

In conclusion, the herein reported hybrid PLGA-phospholipid-targeted formulations showed improved cellular uptake compared to control NPs and good relaxometric properties, highlighting their potential not only as a noninvasive diagnostic tool but also for improving eventual drug delivery efficiency. While this study focused on 2D cell cultures, future research should incorporate 3D tumor spheroid or organoid models to better replicate the TME. Such models can provide insight into NP penetration and uptake in conditions closer to in vivo. Future studies will aim to validate these findings in vivo and explore therapeutic applications using hybrid NPs in advanced tumor models.

## Supplementary Material



## Notes

Polyglutamine synthesis on a solid phase and characterization, 1H NMR spectrum acquisition of polyglutamine, and hemolysis test, assessment of stability of NPs, hemolysis, confocal microscopy of MiaPaca2 cells, confocal microscopy of Panc1 cells, schematic model of the functionalized NPs with a combination of PLGA and PEGylated phospholipid, chemical structures of reagents and PLGA NP preparation steps, scheme of glut SPPS synthesis, data about NP properties, and supplementary references (PDF)

## Notes

FOE contribution to the EuroBioImaging MultiModal Molecular Imaging Italian Node (www.mmmi.unito.it) is gratefully acknowledged.

## Abbreviations

BSA: bovine serum albumin
CE %: conjugation efficiency percentage
DLS: dynamic light scattering
DMEM: Dulbecco’s modified Eagle medium
DMF: *N*,*N*′-dimethylformamide
EPR: enhanced permeability and retention
FBS: fetal bovine serum
GBCA: gadolinium-based contrast enhancement agent
Gd: gadolinium
HBS: HEPES-buffered saline
ICP: inductively coupled plasma
MDCT: multidetector computed tomography
MRI: magnetic resonance imaging
MTT: thiazoly blue tetrazolium bromide
NHS: *N*-hydroxysuccinimide
NMRD: nuclear magnetic relaxation dispersion
NP: nanoparticle
PBS: phosphate-buffered saline
PEG: polyethylene glycol
PLGA: poly­(lactic-*co*-glycolic acid)
PVA: poly­(vinyl alcohol)
SEM: standard error of the mean
*T*_1_: longitudinal relaxation time
