# Supplementary material for: Cellular Uptake of Hybrid PLGA-Lipid Gadolinium Nanoparticles Functionalized for Magnetic Resonance Imaging of Pancreatic Adenocarcinoma Cells
Source: ACS Nanosci Au. 2025 Apr 24;5(3):184–95. doi: 10.1021/acsnanoscienceau.5c00010 (PMC12183577; doi:10.1021/acsnanoscienceau.5c00010)
Supplement: Supplementary file 1 [file ng5c00010_si_001.pdf]

Supplementary information

Cellular Uptake of Hybrid PLGA-Lipid Gadolinium Nanoparticles Functionalised for MRI Imaging of Pancreatic Adenocarcinoma Cells

Alessandro Amaolo [a], Hanieh Sadeghi[a], Carla Carrera[a], Sergio Padovan[a], Fabio Carniato[b], Enza Di Gregorio [a] and Giuseppe Ferrauto [a,*]

[a] *Molecular Imaging Center, Department of molecular biotechnologies and health sciences, University of Torino, Via Nizza 52, 10126 Torino, Italy*

[b] *Dipartimento di Scienze e Innovazione Tecnologica, Università degli Studi del Piemonte Orientale, Via Teresa Michel 11, 15121 Alessandria, Italy*

Content

- Supplementary materials and methods
  - Polyglutamine Synthesis on a Solid Phase and Characterisation
  - ^{1}H-NMR spectrum acquisition of Polyglutamine
  - Haemolysis test
- Supplementary figures, schemes and tables
  - *Figure S1. Assessment of stability of NPs by measuring ζ-potential by DLS, at 25 °C, over a course of 3 days in HBS and compared to control PLGA group.*
  - *Figure S2* Haemolysis upon incubation of Red Blood Cells in the presence of PLGA-NPs (80 μM, 30 min of incubation).
  - *Figure S3.* Confocal microscopy images of MiaPaca2 cells treated with Gd-loaded PLGA NPs.
  - *Figure S4.* Confocal microscopy images of Panc1 cells treated with Gd-loaded PLGA NPs.
  - *Scheme S1. Schematic model of the functionalized nanoparticles with a combination of PLGA and PEGylated phospholipid.*
  - *Scheme S2: Chemical structures of reagents and PLGA NP preparation steps.*
  - *Scheme S3. Scheme of Glut SPPS synthesis using a 2CTC resin*
  - *Table S1. Data about NPs properties*
- Supplementary references

Supplementary materials and methods

Haemolysis test

To evaluate the potential *in vivo* availability, the biocompatibility of NPs was assessed in vitro using the haemolysis assay to determine their impact on red blood cells (RBCs).

Haemolysis assay was performed on RBCs collected from the tail vein of 14–16-week-old male BALB/c mice weighing approximately 25 ± 3 g, using a 27-gauge syringe preloaded with heparin. Blood was diluted in fresh PBS and centrifuged at 2300 rpm for 8 minutes to pellet the cells. The RBCs were washed, recentrifuged, and then exposed to NPs at concentrations of Gd of 80 μM for 30 minutes at room temperature. Post-incubation, samples were centrifuged, and the supernatant was collected to measure the released haemoglobin spectrophotometrically at 413 nm (Soret's band) using a 6715 UV/Vis Spectrophotometer (JEOL). RBCs incubated in fresh PBS served as controls. RBCs lysed using milliQ water were used as positive control, for calculating the total amount or released Hb. The percentage of haemolysis was calculated by using the following formula:

Haemolysis % = $((Abs^{T} - Abs^{ctrl}) / (Abs^{FR})) \times 100$

Where Abs^{T} is the mean absorbance (λ = 413 nm) of treated RBCs and Abs^{ctrl} is the mean absorbance of control RBCs treated with fresh PBS (negative control) and Abs^{FR} is the mean absorbance of RBCs totally lysed by osmotic shoch (incubation in milliQ water 1:6 *v/v*, osmolarity of *ca*. 50 mOsm/L). Cells experiments were repeated in triplicate and data reported as mean ± standard deviation. Blank was repeated 5 times.

Polyglutamine Synthesis on a Solid Phase and Characterisation

Solid phase peptide synthesis (SPPS) was employed to produce the peptide containing 6 glutamine residues (poly(Q6)). The synthesis was conducted on the Liberty Blue automated microwave peptide synthesiser (CEMCorp. Matthews, NC, USA). The synthesis employed the teabag method at a 0.1 mmol scale, utilizing 2-chlorotrityl chloride (2CTC) resin Iris Biotech swelled in N,N'-dimethylformamide (DMF) (1.6 meq/g), to achieve an acidic carboxyl (-COOH) terminal. Single coupling cycles were conducted to ensure efficient assembly of the peptide. Fmoc-Gln (Trt)-OH glutamine reagent (0.2 M), DMF as main solvent (0.01 mmol), Piperidine 20% as deprotection solution, and N,N'-diisopropylcarbodiimide (DIC)/Oxyma (0.5 M, 1 M, respectively) were employed as an activator. Reagent amounts were calculated using Liberty

Blue software. Fmoc-Glutamine (Trityl)-OH (Fmoc-Gln (Trt)-OH) was used as the building block for each coupling reaction. The peptide synthesis proceeded with the Fmoc (9-fluorenylmethyloxycarbonyl) strategy. The resin was initially loaded with the C-terminal glutamine residue, followed by the sequential addition of the remaining five glutamine residues. Each coupling reaction utilised N,N'-diisopropylcarbodiimide (DIC) and Oxyma Pure as activators, dissolved in N,N-dimethylformamide (DMF), to achieve high coupling efficiency. Fmoc deprotection was carried out using 20% piperidine in DMF. Final cleavage was done with a cocktail of TFA/TIS/Water of 95:2.5:2.5 (Trifluoroacetic, triisopropylsilane) for peptide, which was added to dry resin and mixed for 2 hours on a shaker. Peptide precipitation was conducted using liquid-liquid extraction with cold Diethyl ether (80 mL), then dissolved in water and finally lyophilised. Purification and characterization of the peptide were done using High-Performance Liquid Chromatography (HPLC) (JASCO Corporation, Tokyo, Japan) and Mass spectroscopy. Peptides were analysed using reverse-phase high-performance liquid chromatography (RP-HPLC). The analysis employed a gradient of 0% to 70% of solvent B (acetonitrile with 0.1% TFA) against solvent A (water with 0.1% TFA) on an XBridge™ BEH C18 column (100 × 4.6 mm, 3.5 μm) (Waters Corp., Milford, MA, USA) at a flow rate of 0.2 mL/min for 9 minutes.

^{1}H-NMR spectrum acquisition of Polyglutamine

Synthesised polyglutamine was characterised using ^{1}H-NMR spectroscopy. The ^{1}H-NMR spectra of Polyglutamine (15 mg/mL in D_2O or in H_2O) were acquired using a Bruker Avance 400 MHz spectrometer with high-resolution capabilities. The instrument was equipped with 5 mm probes and used D_2O and H_2O as an internal lock at 298 K. The experimental settings included a spectral width of 50 ppm, 256 scans, 4 dummy scans, automatic receiver gain (RG), and a recycle delay of 3 s to ensure optimal peaks integration.

Supplementary figures and schemes

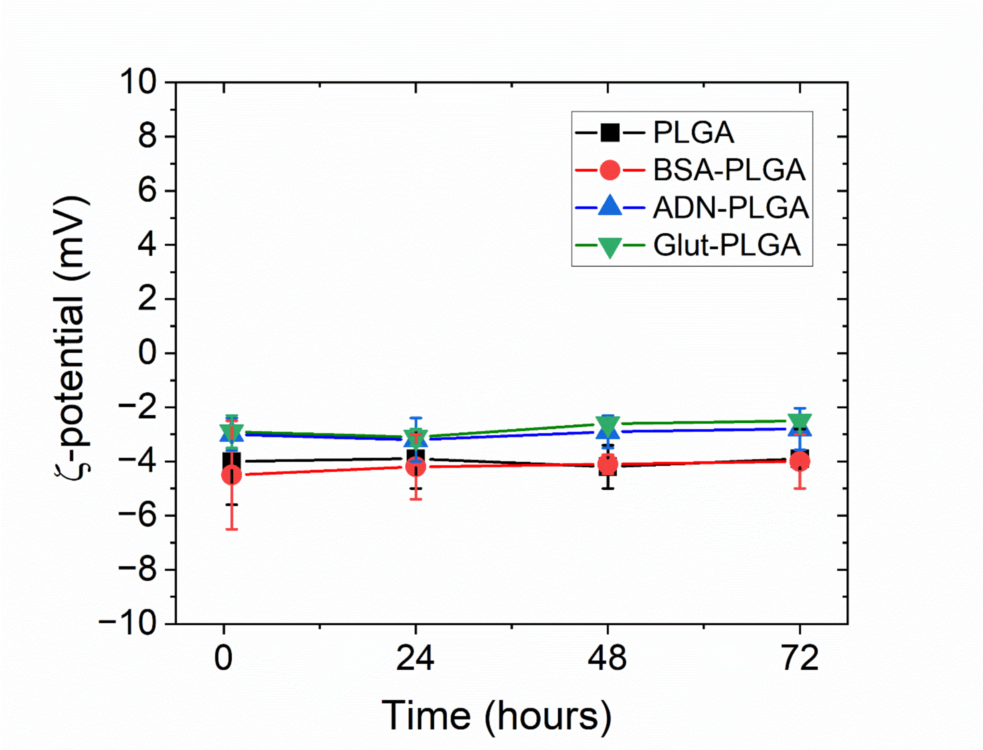

Figure S1 Targeted PLGA-NPs' ζ-potential stabilities were measured by DLS, at 25 °C, over a course of 3 days in HBS and compared to control PLGA group.

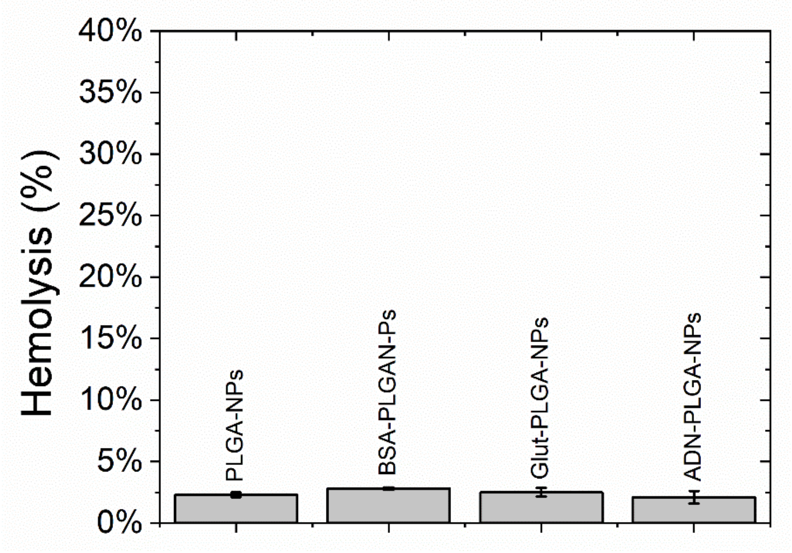

Figure S2 Haemolysis upon incubation of Red Blood Cells in the presence of i) PLGA-NPs, ii) BSA-PLGA-NPs,iii) Glut-PLGA-NPs or iv) AND-PLGA-NPs (80 μM, 30 min of incubation).

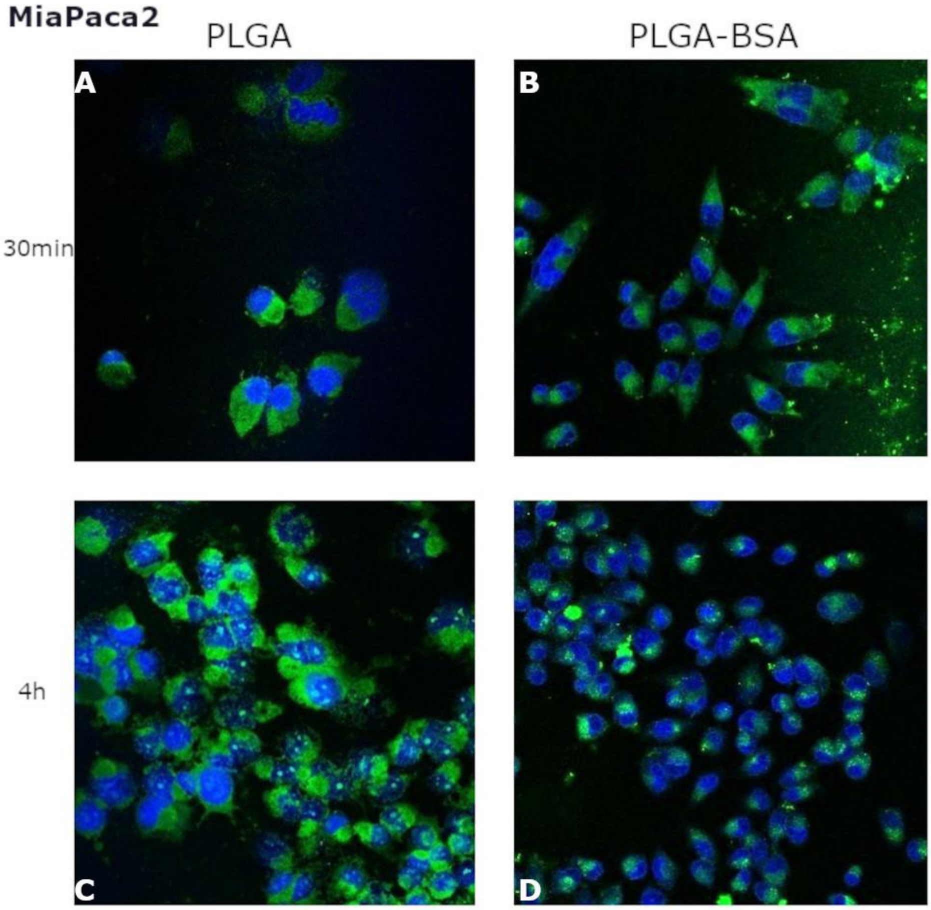

Figure S3. Confocal microscopy images of MiaPaca2 cells treated with Gd-loaded PLGA NPs. (A) Cells treated with non-targeted PLGA NPs after 30 minutes; (C cells treated with non-targeted PLGA NPs after 24 hours; (B) cells treated with BSA functionalised PLGA NPs after 30 minutes; (D) Cells treated with BSA functionalised PLGA NPs after 24 hours. Green fluorescence indicates the presence of NPs, while blue fluorescence corresponds to nuclear staining (DAPI). Scale bars represent 500 µm.

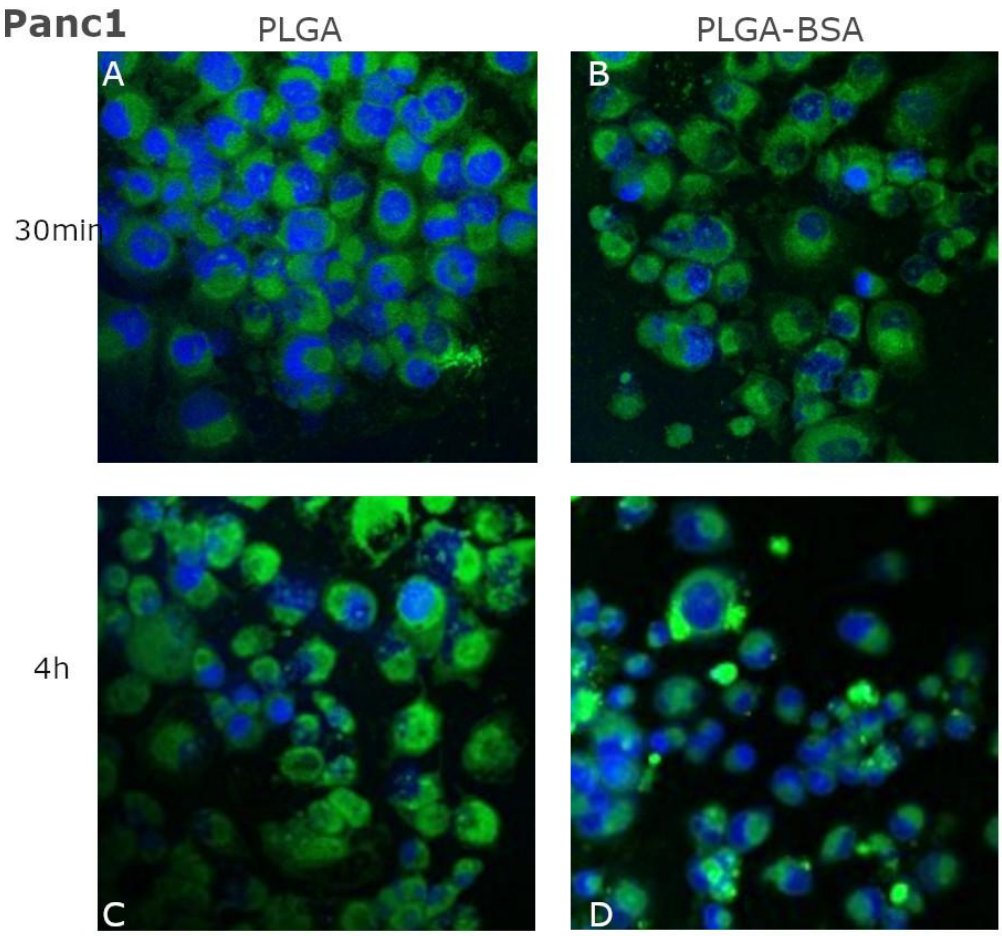

Figure S4. Confocal microscopy images of Panc1 cells treated with Gd-loaded PLGA NPs. (A) Cells treated with non-targeted PLGA NPs after 30 minutes; (C cells treated with non-targeted PLGA NPs after 24 hours; (B) cells treated with BSA functionalised PLGA NPs after 30 minutes; (D) cells treated with BSA functionalised PLGA NPs after 24 hours. Green fluorescence indicates the presence of NPs, while blue fluorescence corresponds to nuclear staining (DAPI). Scale bars represent 500 μm.

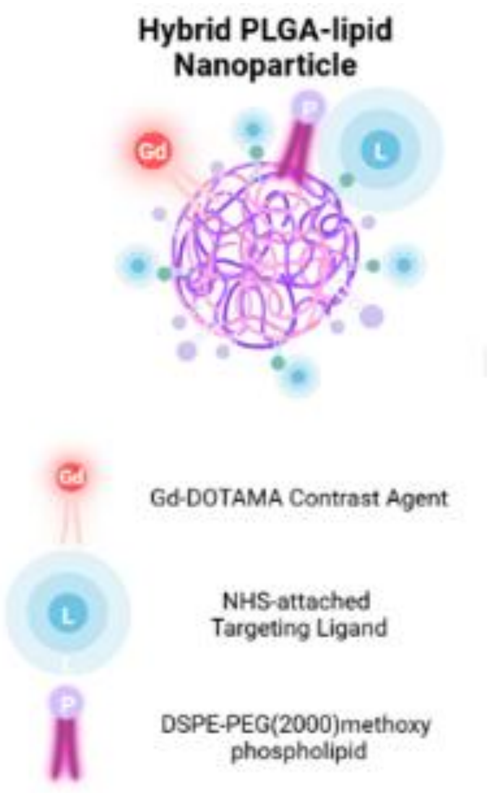

Scheme S1. Schematic model of the functionalized nanoparticles with a combination of PLGA and PEGylated phospholipid.

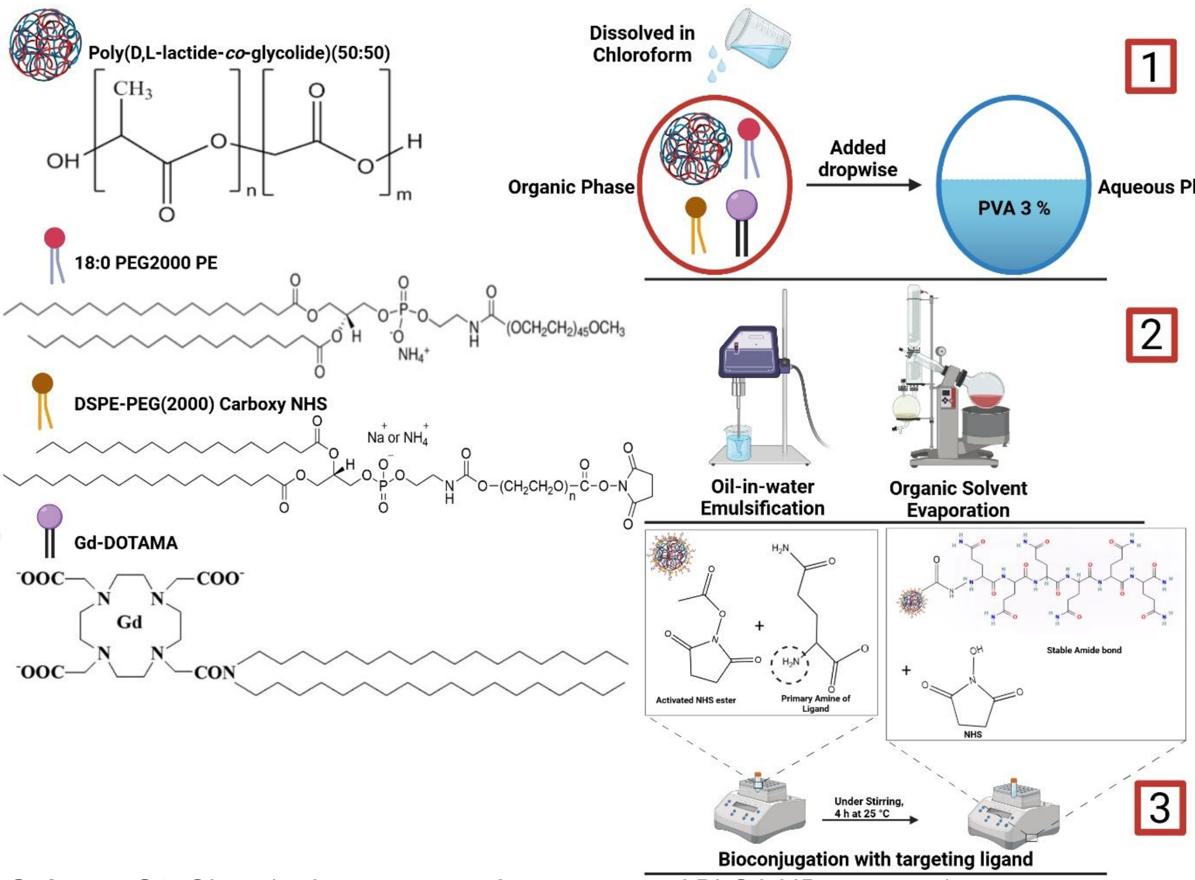

Scheme S2. Chemical structures of reagents and PLGA NP preparation steps.

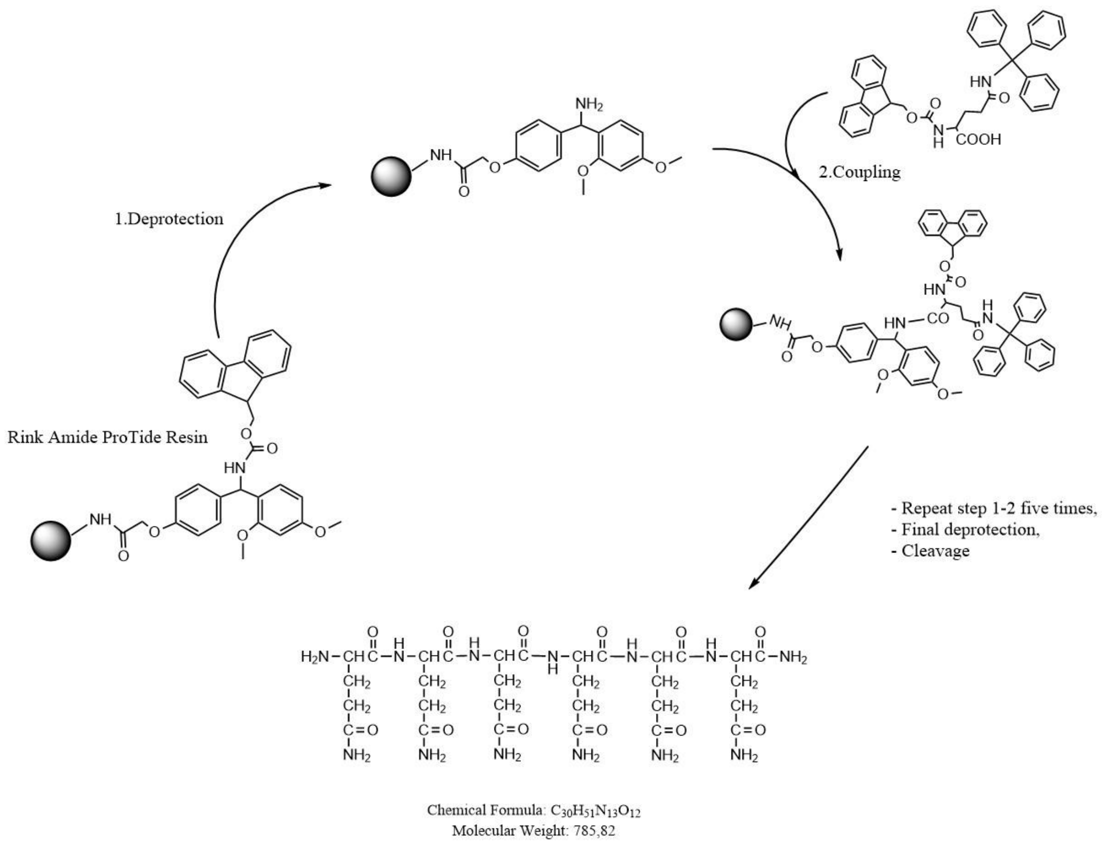

Scheme S3. Scheme of Glut SPPS synthesis using a 2CTC resin

	PLGA	BSA-PLGA	ADN-PLGA	Glut-PLGA
**Particle diameter (nm)**	140 ± 6	155 ± 5	146 ± 11	149 ± 15
**ζ (mV)**	-4.0 ± 1.6	-4.5 ± 2	-3.0 ± 0.6	-2.9 ± 0.6
**polydispersity index (PDI)**	0.053	0.148	0.042	0.123
**Relaxivity (r_{1p}) (mM^{-1} s^{-1}), 21.5 MHz, 25 °C**	23.9± 1.9	27 ± 2.8	22.1 ± 1.6	23.2 ± 2.4
**Gd-complex encapsulation efficiency (%)**	87	88	80	82
**Ligand-to-NP number**	n.a.	3.5 x 10^6	2.6 x 10^6	5.2x 10^6
**CE % of ligand**	n.a.	77	65	99

Table S1. Hydrodynamic particle diameter measured by DLS at 25°C in filtered HBS with their respective Polydispersity Index (PDI), ζ-potential (mV), Millimolar Relaxivities (r_{1p}, mM^{-1} s^{-1}), EE % of the Gd-complex, ligand molecules-to-NP and ligand CE %.

Supplementary references

1 S. K. Sahoo, J. Panyam, S. Prabha and V. Labhasetwar, *Journal of Controlled Release*, 2002, **82**, 105–114.

2 S. K. Hobbs, W. L. Monsky, F. Yuan, W. G. Roberts, L. Griffith, V. P. Torchilin and R. K. Jain, *Proceedings of the National Academy of Sciences of the United States of America*, 1998, **95**, 4607–4612.
